# Annual acknowledgement of manuscript reviewers

**DOI:** 10.1186/s12992-015-0089-9

**Published:** 2015-02-27

**Authors:** Greg Martin

**Affiliations:** UNITAID, World Health Organization, 20 Avenue Appia, Geneva, 27 1211 Switzerland

## Abstract

The editor of *Globalization and Health* would like to thank all our reviewers who have contributed to the journal in Volume 10 (2014).

**Koki Agarwal**

United States of America

**Charles Alessi**

United Kingdom

**Marcella Alsan**

United States of America

**Atsuko Aoyama**

Japan

**Daniel Arhinful**

Ghana

**Phillip Baker**

Australia

**Amy Barnes**

United Kingdom

**Simon Barraclough**

Australia

**David Beran**

Switzerland

**Ramesh Bhat**

India

**Vivian Black**

South Africa

**Katherine Bliss**

United States of America

**Gerald Bloom**

United Kingdom

**Chantal Blouin**

Canada

**Hannah Brinsden**

United Kingdom

**Joshua Busby**

United States of America

**Meena Cabral de Mello**

Switzerland

**Guido Calleri**

Italy

**Padraig Carmody**

Ireland

**Francesco Castelli**

Italy

**Lai-Ha Chan**

Australia

**Matthew Chersich**

South Africa

**Enrica Chiappero**

Italy

**Maulik Chokshi**

India

**Heather Cole-Lewis**

United States of America

**Adrijana Corluka**

Canada

**Laura Cornelsen**

United Kingdom

**Valorie Crooks**

Canada

**Viva Dadwal**

Canada

**Mario Roberto Dal Poz**

Brazil

**Karen Daniels**

South Africa

**Saeed Dastgiri**

Iran

**Jose A. Diaz Huertas**

Spain

**Joseph Dieleman**

United States of America

**Kate Elder**

United States of America

**Charles Evans**

United States of America

**Victoria Fan**

United States of America

**Laura Ferguson**

United States of America

**Sonia Fevre**

United Kingdom

**James Fitzgerald**

United States of America

**Ruth FItzgerald**

New Zealand

**Mohammad H. Forouzanfar**

United States of America

**Paul Freeman**

United States of America

**Michelle L. Gagnon**

Canada

**Chandika Gamage**

Japan

**Annette Ghee**

United States of America

**Brynne Gilmore**

Ireland

**Amanda Glassman**

United States of America

**Jeff Gow**

South Africa

**Paul Grootendorst**

Canada

**Sofia Gruskin**

United States of America

**Rachel Hammonds**

Belgium

**Sophie Harman**

United Kingdom

**Emily Jacqueline Henderson**

United Kingdom

**Sarah Hepworth**

United Kingdom

**Cristina Hernandez Quevedo**

United Kingdom

**Heikki Hiilamo**

Finland

**Jarno Hoekman**

Netherlands

**Aidan Hollis**

Canada

**Simone Honikman**

South Africa

**Weng foung Huang**

Taiwan

**Suzanne Jackson**

Canada

**Kathryn H. Jacobsen**

United States of America

**Jordan Jarvis**

United States of America

**Jo Jewell**

United Kingdom

**Michel Joffres**

Canada

**Helle Johannessen**

Denmark

**Rory Johnston**

Canada

**Andrew Jones**

United Kingdom

**Christine Jowi**

Kenya

**Kiti Kajana**

United States of America

**Warren Kaplan**

Switzerland

**Warren Kaplan**

Switzerland

**Inge Kaul**

Germany

**Caitlin Kennedy**

United States of America

**Shusmita Hossain Khan**

Bangladesh

**Ilona Kickbusch**

Switzerland

**Regine King**

Canada

**Sandeep Kishore**

United States of America

**Tracy Kline**

United States of America

**Jeroen Klomp**

Netherlands

**Jillian Clare Kohler**

Canada

**Meri Koivusalo**

Finland

**Nancy Krieger**

United States of America

**Ronald Labonte**

Canada

**Lance Laird**

United States of America

**Patrick Lee**

United States of America

**Kelley Lee**

Canada

**David Legge**

Australia

**Jane Lethbridge**

United Kingdom

**Timothy Mackey**

United States of America

**Mats Malqvist**

Sweden

**Greg Martin**

Ireland

**Adriane Martin Hilber**

Switzerland

**Melisa Martínez Álvarez**

United Kingdom

**Duane McBride**

United States of America

**Ted McDonald**

Canada

**Benn McGrady**

United States of America

**Paul Mee**

United Kingdom

**David Merriman**

United States of America

**Charles Mgone**

Netherlands

**Eduardo Missoni**

Italy

**Marc Mitchell**

United States of America

**Erik Monasterio**

New Zealand

**Julia Moorman**

South Africa

**Chester Morris**

Nigeria

**Kathryn Muessig**

United States of America

**Falk Müller-Riemenschneider**

Singapore

**Susan Murray**

United Kingdom

**Bronwyn Myers**

South Africa

**Murugesan N**

India

**Jenneken Naaldenberg**

Netherlands

**Anchana NaRanong**

Thailand

**Thomas Novotny**

United States of America

**David Ofori-Adjei**

Ghana

**Meghann Ormond**

Netherlands

**Ajibike Oyewumi**

Canada

**Aasim Padela**

United States of America

**Stacey Page**

Canada

**Ligia Paina**

United States of America

**Ulysses de Barros Panisset**

Switzerland

**Pierre Emmanuel Paradis**

Canada

**Charles Parry**

South Africa

**Mahomed Patel**

Australia

**Enrico Pavignani**

Mozambique

**Dennis Petrie**

Australia

**Yayi Prabandari**

Indonesia

**Yayi Prabandari**

Indonesia

**Tara Prasad**

United Kingdom

**Judith Proudfoot**

Australia

**Pekka Puska**

Finland

**Michea Quinlan**

Australia

**Tim Quinlan**

South Africa

**Tracy Rabin**

United States of America

**Aaron Reeves**

United Kingdom

**Erica Richardson**

United Kingdom

**Susan Rifkin**

United Kingdom

**Nirmal Rimal**

Nepal

**Montserrat Rue**

Spain

**Simon Rushton**

United Kingdom

**Nancy Sanchez**

Cuba

**Sylvia Sax**

Germany

**Georg Schomerus**

Germany

**Ashley Schram**

Canada

**Alexander Schuhmacher**

Germany

**Maryam Shahmanesh**

United Kingdom

**Hirohisa Shimura**

Japan

**Sameen Siddiqi**

Egypt

**Jeremy Snyder**

Canada

**Neil Spicer**

United Kingdom

**Elizabeth Stierman**

Zambia

**Elizabeth Stierman**

Zambia

**Julie Storr**

United Kingdom

**Kai Straehler-Pohl**

Germany

**Bjørn Heine Strand**

Norway

**Shamsuzzoha Syed**

Switzerland

**Kenichiro Taneda**

Philippines

**Christine Kirunga**

Tashobya Uganda

**Lauren Taylor**

United States of America

**Martin Taylor**

China

**George Thomson**

New Zealand

**Mike Toole**

Australia

**Ruth Townsend**

Australia

**Erika Turkstra**

Australia

**Regina Ungerer**

Switzerland

**Frédérique Vallières**

Ireland

**Wannes Van Hoof**

Belgium

**Mohamud Verjee**

Qatar

**Taryn Vian**

United States of America

**Salvador Villalpando**

Mexico

**Joseph Vinetz**

United States of America

**Helen Walls**

United Kingdom

**Yizhou Wan**

United Kingdom

**David Watkins**

United States of America

**Alan Whiteside**

Canada

**Suwit Wibulpolprasert**

Thailand

**Heather Wipfli**

United States of America

**Albert Wu**

United States of America

**Annalee Yassi**

Canada

**Jeremy Youde**

United States of America

**Lou Yu**

China

**Haiyu Zhao**

China

**Shanshan Zhou**

China

**Cathy Zimmerman**

United Kingdom

**Genyong Zuo**

China

